# Investigation of Cu Adsorption and Migration with Spectral Induced Polarization in Activated Carbon

**DOI:** 10.3390/toxics11030221

**Published:** 2023-02-26

**Authors:** Bate Bate, Jingjing Cao, Yixin Yang, Junnan Cao, Chi Zhang, Shuai Zhang

**Affiliations:** 1 College of Civil Engineering and Architecture, Zhejiang University, Hangzhou 310027, China; 2 Department of Civil Engineering and Construction, Georgia Southern University, Statesboro, GA 30458, USA; 3 Department of Meteorology and Geophysics, University of Vienna, Josef-Holaubek-Platz 2 (UZA II), 1010 Vienna, Austria

**Keywords:** heavy metal, activated carbon, spectral induced polarization, monitor, ion exchange, complex conductivity

## Abstract

In this paper, the adsorption process of copper ions on activated carbon (AC) was simulated in a column test. It was deduced that it is consistent with the pseudo-second-order model. Cation exchange was observed to be the major mechanism of Cu–AC interactions through scanning electron microscopy–energy dispersive X-ray spectroscopy (SEM–EDS), X-ray diffraction (XRD), and Fourier transform infrared spectroscopy (FTIR) measurements. Adsorption isotherms were fitted well using the Freundlich model. Adsorption thermodynamics at 298, 308, 318 K demonstrated that the adsorption process is spontaneous and endothermic. Spectral induced polarization (SIP) technique was used to monitor the adsorption process, and the double Cole–Cole model was used to analyze the SIP results. The normalized chargeability was proportional to the adsorbed copper content. Two measured relaxation times from the SIP testing were converted into the average pore sizes of 2, 0.8, 0.6, 100–110, 80–90, and 53–60 µm by the Schwartz equation, which are consistent with the measured pore sizes from both mercury intrusion porosimetry and scanning electron microscopy (SEM). The reduction in the pore sizes by SIP during the flow-through tests suggested that the adsorbed Cu^2+^ gradually migrated into small pores as with continued permeation of the influent. These results showcased the feasibility of using SIP technique in engineering practice involving the monitoring of copper contamination in land near a mine waste dump or in adjacent permeable reactive barriers.

## 1. Introduction

Heavy metal contamination is a vital environmental concern in most developing countries, causing soil and air pollution, disrupting ecosystems, and threatening human health. Copper, which is one of the most common heavy metals, is primarily derived from industrial wastewater [1], mineral processing [2], agricultural practices [3], and landfills [4]. Groundwater contaminated by Cu and other heavy metals can be contained and remediated using permeable reactive barriers (PRBs), which were used in several studies and led to satisfactory results [5,6,7,8]. Activated carbon (AC), which is one of the most common barrier materials, is frequently used as a cost-effective adsorbent for pollutants, including heavy metals and dissolved organic and inorganic species [9].

The adsorption mechanism of AC is determined by its physical properties (i.e., pore size distribution and specific surface area) and the nature of its surface chemicals (i.e., functional groups) [10]. The mechanisms include surface complexation, electrostatic interaction, physical adsorption, and ion exchange [11,12]. Rio [13] demonstrated that the removal of Cu(II) using biochar was dominated by ion exchange with Ca(II) on the biochar surface. Xie [11] showed that ion exchange may be considered the main mechanism in the adsorption of Cu(II) on the AC surface, and the carboxyl and hydroxyl groups are mainly responsible for this process. However, to the best of our knowledge, the dynamic evolution during the adsorption process has not been revealed yet. Real-time monitoring of heavy metal remediation should be further studied.

Emerging electrical geophysical methods have been widely used to study the hydraulic–physical–chemical properties and reaction process of porous media, such as direct-current (DC) electrical resistivity, induced polarization (IP), and spectral induced polarization (SIP). The latter is a promising, real-time, and nondestructive technique that can reveal the electrical properties of a porous medium to reflect its characterization information and monitor its physical–chemical–biological interaction processes at low frequency (i.e., in the range of 0.01–1000 Hz). Compared with other existing methods, such as DC electrical resistivity and IP, SIP provides information on the electrical properties of the porous media, which demonstrates electrical conduction (charge carrier electromigration) and polarization effects (energy storage) in the real and imaginary parts of the complex conductivity [14,15]. SIP has been widely used to study microbial growth and biofilm formation [16,17,18], biochar remediation and characterization [19,20,21], calcite precipitation [22,23], and organic pollution [24,25]. It also has high potential in the in situ detection and monitoring of contaminant transport, chemical reactions due to the sensitivity to interfacial properties (i.e., ionic mobility and surface charge density) of porous media, and conditions of the aqueous phase such as the salinity, saturation, and temperature. Thus, the application of SIP has high potential for monitoring the remediation process.

The cationic contaminants remediated by porous media have strong SIP signatures, which makes real-time monitoring by SIP feasible. Several studies monitored the adsorption of contaminants using porous materials. For instance, Masi et al. [26] studied the electrokinetic remediation processes of heavy metal-contaminated fine-grained marine sediments using SIP. They deduced that pH is one of the most important parameters affecting the polarization mechanism. In addition, they observed a linear link between the total chargeability and the pH. Ben Moshe et al. [27] performed SIP on a soil sample to demonstrate the ion exchange process (Na^+^-Ca^2+^, Na^+^-Zn^2+^). This was strongly related to ion mobility and Stern layer polarization, and it was reflected through the variation of the imaginary conductivity. Hao et al. [28] presented the processes of Pb^2+^ and Cd^2+^ flow-through on loess using SIP. They differentiated the adsorption mechanisms between loess and Pb^2+^/Cd^2+^ using SIP signals and correlated the decrease of the imaginary conductivity and reduced total polarizable surface charges with the shift of the characteristic frequency and calcite dissolution. Siddiq et al. [29] studied the process of SIP detection in biochar-based As remediation. They deduced that the real and imaginary components will be affected by the amount of As adsorption caused by the interaction with surface functional groups. To the best of our knowledge, research on the SIP responses of the AC-based Cu remediation process is scarce. In addition, knowledge of the mechanisms of Cu–AC interaction evolution during the adsorption process incorporating SIP technology is still lacking.

The goal of this study is to innovatively incorporate the SIP method in monitoring the process of Cu^2+^ adsorption onto AC in a column test setup. The objectives are as follows: (1) Perform a flow-through test under 1, 5, and 10 mmol/L CuCl_2_ concentrations with SIP monitoring, (2) Perform thermodynamic experiments and propose a kinetic equation, (3) Perform microscopic and physicochemical experiments to elucidate the fundamental mechanisms of Cu–AC interactions, (4) Examine the SIP signals and analyze correlations to the quantity and the signature sizes of the Cu–AC units, and then evaluate the feasibility of SIP as a monitoring technique in a permeable reactive barrier or in contaminated land near a copper mine waste dump.

## 2. Background of SIP

SIP is a noninvasive geophysical method for measuring the electrical properties and polarization phenomena of porous media. It can be expressed by the complex conductivity ($\sigma^{*}$) or resistivity ($\rho^{*}$), which is its inverse. The complex conductivity is expressed as [30,31]:

(1) $$\sigma^{*}(\omega )=\frac{1}{\rho^{*}}=|\sigma (\omega )|\exp(i\phi )=\sigma^{\prime }(\omega )+i\sigma^{\prime \prime }\left(\omega \right)$$

where $\sigma^{\prime }$(S/m) is the real or in-phase conductivity representing the energy loss of charge carrier electromigration, $\sigma^{\prime \prime }$(S/m) is the imaginary or quadrature conductivity representing the energy storage of charge carrier polarization, |σ| is the magnitude of $\sigma^{*}$, ω(rad/s) is the angular frequency, and $i^{2}=-1$ denotes the imaginary number.

Three main mechanisms (Stern layer, diffuse layer, and membrane polarizations) were observed in the SIP measurements that are closely related to the dispersion of the complex conductivity caused by the polarization of the electrical double layer (EDL) at the solid–liquid interface at a low frequency (<1 MHz) [32]. When an external alternating electrical field is applied, the counterions in the EDL move in the direction of the electric field and accumulate at the edge of the grain. Once the injected current is shut off, the charges will balance to the previous status through ion diffusion [30].

To evaluate the performance of the SIP method in monitoring the copper ion adsorption process on AC at CuCl_2_ concentrations ranging between 1 mmol/L and 10 mmol/L, the following tasks were conducted: (1) Chemical analytical techniques were performed to study the possible mechanisms of copper adsorption on AC. A combination of breakthrough column experiments and an adsorption kinetic model was performed to predict and reflect the evolutions of the geochemical process with time. (2) The real and imaginary conductivities were measured using SIP to study the copper adsorption mechanism. (3) The double Cole–Cole model was used to fit the SIP experiment data for comparison and further discussion.

The SIP data acquired from experiments mostly follow a Cole–Cole distribution and have been widely analyzed with the Cole–Cole model in resistivity or conductivity forms. In this study, the Cole–Cole model was used in its conductivity form and an overlay of two individual Cole–Cole terms according to the bimodal SIP response. The double Cole–Cole model is expressed as:

(2) $$\sigma^{*}(\omega )=\sigma_{0}\left[1+\frac{m_{1}}{1-m_{1}}\left(1-\frac{1}{1+{\left(i\omega \tau_{1}\right)}^{c_{1}}}\right)+\frac{m_{2}}{1-m_{2}}\left(1-\frac{1}{1+{\left(i\omega \tau_{2}\right)}^{c_{2}}}\right)\right]$$

where $m=\left(\sigma_{\infty }-\sigma_{0}\right)/\sigma_{\infty }$ is the chargeability [33,34,35], $\sigma_{0}$ and $\sigma_{\infty }$ (S/m) are respectively the electrical conductivities at low (DC) and high frequencies, $\tau_{1}$ and $\tau_{2}$ are the time constants, and $c_{1}$ and $c_{2}$ are the Cole–Cole exponents that reflect the broadness of the relaxation time distribution [36]. 

For simplification, the normalized chargeability ($m_{n}=m\sigma_{\infty }=\sigma_{\infty }-\sigma_{0}$), which is equal to the conductivity difference at high-limit and low-limit frequencies, is widely used to quantify the degree of polarization [34,37,38]. 

According to the Schwartz equation [31], the average particle size (*d*) in the porous media can be calculated from the peak frequency ($f_{peak}$) as:

(3) $$\tau =\frac{d^{2}}{8D_{\left(+\right)}^{S}}=\frac{1}{2\pi f}$$

where τ (s) is the time constant and $D_{\left(+\right)}^{S}$ ($m^{2}s^{-1}$) denotes the diffusion coefficient of counterions in the Stern layer, which is related to the mobility of the counterions in the Stern layer based on the Nernst–Einstein relation ($D_{\left(+\right)}^{s}=k_{b}T\beta_{\left(+\right)}^{s}/e$). 

The ion mobility in the Stern layer is a reduction of the corresponding value in the diffuse layer by a factor of 10 ($\beta_{\left(+\right)}^{s}=\beta_{\left(+\right)}^{d}/10$). In other words, $D_{\left(+\right)}^{S}=D_{\left(+\right)}^{D}/10$, where $D_{\left(+\right)}^{D}$ is the diffusion coefficient in the diffuse layer.

## 3. Materials and Methodology

AC samples made from coconut shells with particle sizes in the range of 1–2 mm were provided by the Henan province of China and produced by physical steam activation at 1000 °C. The sample was directly rinsed with deionized (DI) water until the electrical conductivity of the supernatant became less than 100 μS/cm. In addition, air bubbles residing in activated carbon were removed to ensure full saturation by DI water.

The mercury intrusion porosimetry (MIP) (Micromeritics, AutoPore IV 9510) was used to measure the pore size distribution, and multipoint Brunauer–Emmett–Teller (BET) specific surface area of the samples was analyzed by nitrogen adsorption (Quantachrome, Autosorb-iQ2-mp). SEM and energy-dispersive spectroscopy (EDS) were used to observe the morphology and elemental composition of the surface using an FEI Nova 450 and Raith Elphy Quanta. X-ray diffraction (XRD) and FTIR spectroscopy were performed to obtain the crystallographic information and functional groups of the AC samples using an Escalab 250Xi spectrometer and a Thermo Scientific Nicolet iS50 FTIR.

The copper adsorption performance of AC was analyzed through batch experiments. About 10 g of AC samples was added to 250mL of a copper solution with concentrations from 20 to 700 mg L^−1^ and shaken at a rate of 110 rpm in a constant temperature shaker incubator. The adsorption kinetics experiments were conducted to study the factor of contact time (5, 10, 20, 30, 40, 60, 80, 130, 200, and 300 min) at 298 K with Cu(II) concentration of 60, 300, 700 mg L^−1^. The adsorption isotherms experiments were performed to investigate the effect of the initial concentrations (20, 40, 60, 100, 200, 300, 500, and 700 mg L^−1^) at the temperatures of 298, 308, 318 K. The Erlenmeyer flasks were kept shaking for 24 h to achieve equilibrium. The influence of temperature was analyzed based on the adsorption isotherms experiments.

**Figure 1:**
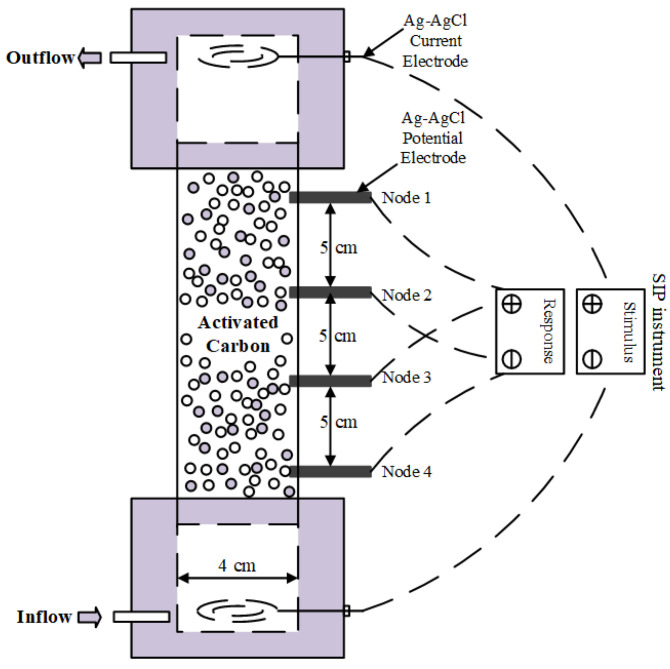
SIP column for the flow-through experiment.

The column flow-through test was conducted as follows. The AC samples were first transferred into a SIP column of size 210 × 40 × 10 mm (length × inner diameter × thickness) using the wet packing filling method, as shown in Figure 1. DI water was then injected into the sample with a peristaltic pump through the bottom of the column until it was fully immersed. Afterwards, three sets of experiments were performed, in which CuCl_2_ solutions having concentrations of 1, 5, and 10 mmol/L were injected into the columns for 60, 30, and 30 h, respectively. During the fluid injections, SIP signals were measured using the PSIP instrument (Ontash & Ermac, Inc., River Edge, NJ, USA) within a frequency range of 0.01–1000 Hz (Figure 1). Two pairs of electrodes were used to measure the potentials of upper (Node 1–2) and bottom (Node 3–4) of the column. The obtained complex conductivities were then further analyzed through Equations (1)–(3) in Section 2. A constant flow rate of 1 mL/min was maintained, and the temperature was set to 25 °C. Finally, the outflow solutions were collected using an automatic collection device, and the concentrations of metal ions were measured by ICP-OES (Avio 220 Max ICP-OES Scott).

## 4. Results and Discussion

### 4.1. Characterization of AC Samples

**Figure 2:**
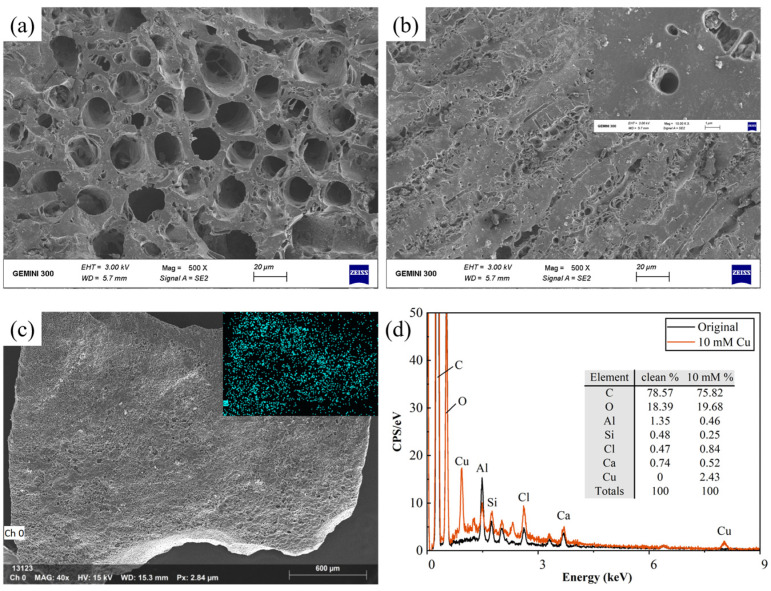
SEM images of the (**a**,**b**) pores on the pure AC. (**c**) SEM–EDS images of AC contaminated by 10-mM Cu. (**d**) EDS analysis of pure and contaminated AC samples.

**Figure 3:**
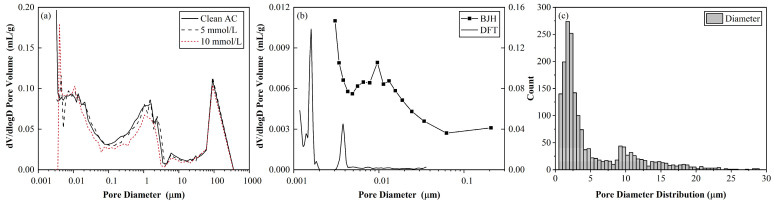
Pore size distribution of (**a**) clean and polluted AC during 5-mmol/L adsorption using MIP and (**b**) clean AC sample using BJH and DFT measurements (represented by the left and right vertical axes, respectively). (**c**) ImageJ analysis of SEM results for the clean AC sample.

*SEM–EDS*. The images of uncontaminated AC (Figure 2) show honeycomb pores with two main pore sizes of approximately 1 μm and 20 μm (Figure 3a,b, respectively). After the Cu^2+^ contamination, Cu spots were evenly distributed on the AC surface, as shown in Figure 2c. The EDS results show spikes in the magnitudes of Cu and Cl. The weight percentage of the chemical element constituents (Figure 2d) shows a decrease in Al (1.35%–0.46%) and Ca (0.74%–0.52%) and an increase in Cu (0%–2.43%). This indicates that Ca^2+^ was displaced by Cu^2+^, most probably through a cation exchange reaction [39], and no precipitation or crystallization occurred.

*BET and MIP*. The surface areas calculated using the BET and MIP methods were 132.653 m^2^/g and 230 m^2^/g, respectively. The pore size distribution of the AC sample obtained using MIP was trimodal, with major peaks at approximately 0.01, 1, and 100 μm, which did not change after the Cu(II) uptake, as shown in Figure 3a. BJH and DFT methods were employed to determine the mesopore and microspore distributions in Figure 3b. Obvious peak at 0.01 μm can be observed, which corresponds to the results in MIP methods (Figure 3a). ImageJ was used for the pore size distribution analysis on the SEM images of the AC (Figure 3c). This indicates the limited copper crystals or precipitate generation. The analysis results of ImageJ are consistent with the MIP results. That is, 1 μm and 10 μm were the dominant pore diameters, as shown in Figure 3.

**Figure 4:**
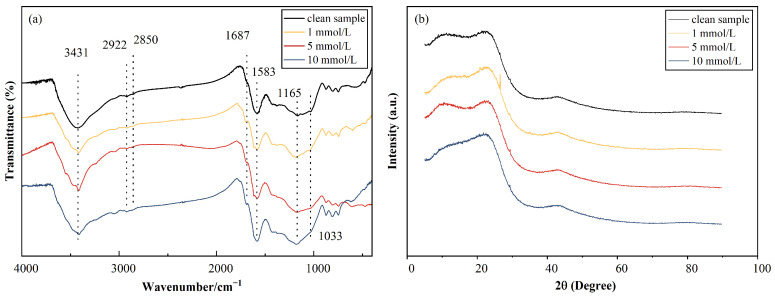
(**a**) FTIR results of AC permeated with CuCl_2_ solutions. (**b**) XRD image of AC at different Cu(II) concentrations.

*FTIR spectroscopy.* Figure 4a shows the FTIR spectroscopy results for the pure and 10-mM contaminated AC within the wavenumber range of 400–4000 cm^−1^. The adsorption at approximately 1033 cm^−1^ and 1165 cm^−1^ was assigned to the C–O stretching in alcohols, phenols, and carboxylic acid groups [40,41]. In addition, a strong bond around 1583 cm^−1^ corresponded to the C=C stretching [41,42]. The peaks around 1687 cm^−1^ were ascribed to the stretching of C=O groups [11]. Moreover, two small peaks of absorption at 2850 cm^−1^ and 2922 cm^−1^ were ascribed to the asymmetric and symmetric C–H stretching, respectively [43] (p. 283). Furthermore, another strong band at approximately 3450 cm^−1^ was attributed to the vibrations of the O–H groups [44]. After the flow-through of Cu, the results of the variations show that three main functional groups (O–H, C=C, and C=O) participated in the Cu(II) adsorption process [12,45].

*XRD.* In the XRD results shown in Figure 4b, two broad peaks at approximately 2θ=24° and 43°, corresponding to the (0 0 2) and (1 0 0) graphitic structures [46], were observed. A small and sharp peak at 2θ=29° representing the CaCO_3_ crystalline phase [47] was detected for the pure and contaminated AC. A sharp peak at 2θ=26.5° was observed in the 1-mmol/L CuCl_2_ sample, which is attributed to the quartz that may originally exist in AC [48].

### 4.2. Adsorption Performance

#### 4.2.1. Adsorption Isotherms

To better understand the adsorption isotherms, Langmuir and Freundlich models are employed to fit the adsorption results by following equations:

(4) $$q_{e}=\frac{K_{L}q_{m}C_{e}}{1+K_{L}C_{e}}$$

(5) $$q_{e}=K_{F}C_{e}^{1/n}$$

where $q_{e}$ and $q_{m}$ (mg g^−1^) are respectively the amounts of copper adsorbed at equilibrium and maximum adsorption capacity, $C_{e}$ (mg L^−1^) is the equilibrium concentration of adsorbate, $K_{L}$ (L g^−1^) and $K_{F}$ (L g^−1^) are Langmuir and Freundlich equilibrium constants, and 1/n is the Freundlich coefficient.

**Figure 5:**
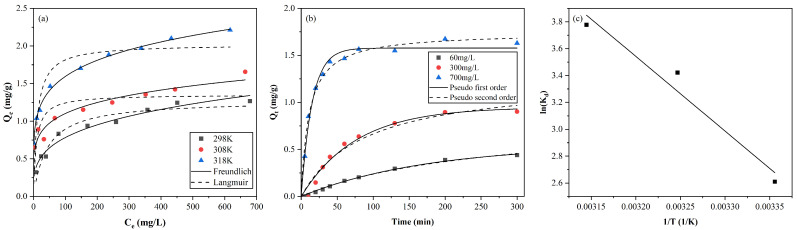
Adsorption (**a**) isotherms at 298, 308, 318 K, and (**b**) kinetic, and (**c**) thermodynamics studies for adsorption of Cu(II) on AC.

Nonlinear fitting results for the adsorption isotherms at 298 K, 308 K, 318 K were plotted in Figure 5a with detailed parameters tabulated in Table 1. The adsorption capacities of AC increased with the initial concentration and gradually reached equilibrium at high concentrations. It is shown that the Freundlich model represents better correlation coefficient than the Langmuir model, suggesting the adsorption process is mainly multilayer. A comparison with the maximum capacity of other adsorbents for copper adsorption is shown in Table 2.

#### 4.2.2. Adsorption Kinetics

The rate-determining step of adsorption process was studied using pseudo-first-order and pseudo-second-order models:

(6) $$\ln\left(q_{e}-q_{t}\right)=-k_{1}t+\ln\left(q_{e}\right)$$

(7) $$\frac{t}{q_{t}}=\frac{1}{k_{2}q_{e}^{2}}+\frac{t}{q_{e}}$$

where $q_{t}$ and $q_{e}$ (mg/g) are respectively the amounts of copper adsorbed at time t and at equilibrium. $k_{1}$ (min^−1^) and $k_{2}$ (g mg^−1^min^−1^) are the adsorption rate constants of the first-order and second-order reactions, respectively.

The adsorption kinetics fitting results of two common models are shown in Figure 5b and the corresponding parameters were tabulated in Table 3. In the existing experiments on the adsorption of copper by activated carbon [49], the pseudo-second-order model exhibits better agreement with experiments results than pseudo-first-order. According to the correlation coefficients (*R*^2^), it can be observed that the two models perform similar fitting results and both well described the kinetics processes.

#### 4.2.3. Adsorption Thermodynamics

The relation thermodynamics parameters, such as the change in enthalpy (ΔH, kJ mol^−1^), entropy (ΔS, kJ K^−1^ mol^−1^), and Gibbs energy change (ΔG, kJ mol^−1^) are calculated as follows:

(8) $$\Delta G=-RT\ln K_{d}$$

(9) $$K_{d}=K_{g}\cdot M\cdot [Adsorbate]{}^{\circ}$$

(10) $$\ln K_{d}=\frac{\Delta S}{R}-\frac{\Delta H}{RT}$$

where $K_{d}$ is the thermodynamic equilibrium constant, $K_{g}$ is constant of the best isotherm model fitted ($K_{F}$ is replaced here), [Adsorbate]° (1 mol L^−1^) is the standard concentration of the adsorbate, M (g.mol^−1^) is the molecular weight of adsorbate, R (8.314 J mol^−1^ K^−1^) is the universal gas constant, and T (K) is the absolute temperature of the solution.

The Van ’t Hoff graph is shown in Figure 5c and values of ΔH and ΔS were obtained from the slope and the intercept of line of $\ln K_{d}$ versus 1/T. Detailed thermodynamic parameters are shown in Table 4. The negative ΔG and positive ΔH indicate that the adsorption process of Cu^2+^ is thermodynamic spontaneous and endothermic, which means that the increase in temperature benefits the adsorption process. This is consistent with the previous results of thermodynamic experiments [49,50]. The positive ΔS indicates that the degree of disorder at the solid–liquid interface increases at higher temperatures, and the AC has high affinity for Cu^2+^ in aqueous solution [51].

**Table 2:** Copper adsorption capacities for different adsorbents.

Adsorbent	Capacity (mg/g)	References
Granular activated carbon	5.845	[52]
Coconut shell-based carbon	7.04	[53]
Orange peel	44.28	[54]
Chitosan-coated perlite bead	104	[55]
Carbon nanofibers	8.8	[56]
Bottom ash of expired drugs incineration	13.335	[57]
Biochar made from pinewood pyrolysis	2.73	[58]
Biochar produced from wood waste	2.9	[59]
Cellulose hydrogel	52.3	[60]
Activated carbon	2.078	This study

**Table 3:** Kinetics parameters of Cu(II) adsorption onto AC.

Kinetics Models	Parameters	Value		
		60 mg/L	300 mg/L	700 mg/L
Pseudo-first-order	q_e_ (mg.g^−1^)	0.546	0.949	1.578
	K_1_ (min^−1^)	0.00579	0.0131	0.06536
	R^2^	0.995	0.969	0.988
Pseudo-second-order	q_e_ (mg.g^−1^)	0.842	1.256	1.744
	K_2_ (g.mg^−1^min^−1^)	0.00467	0.00894	0.05187
	R^2^	0.993	0.957	0.990

### 4.3. Geochemical Process

**Figure 6:**
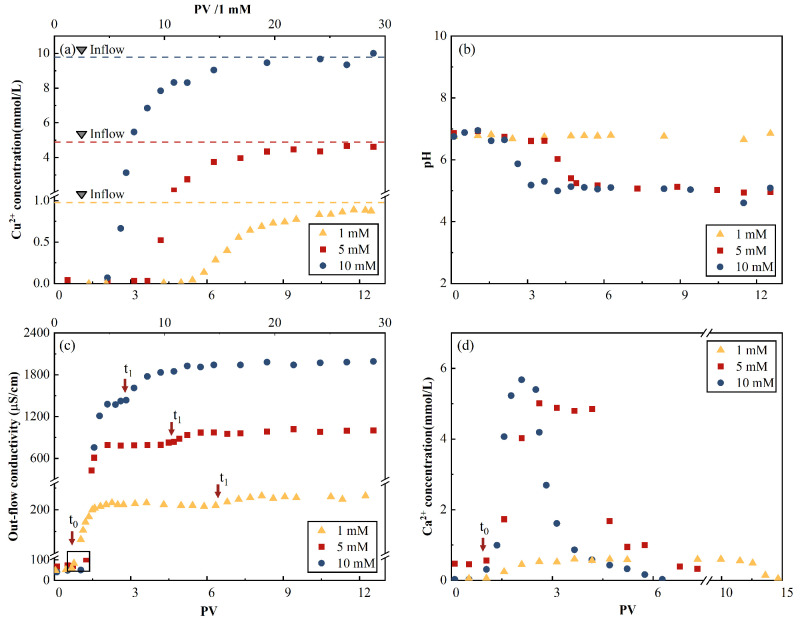
Time evolution of the outflow properties. (**a**) Cu^2+^ concentration, (**b**) pH, (**c**) electrical conductivity, and (**d**) Ca^2+^ concentration. Note: the two-segment *x*-axis scale is used for a better illustration of the results for the 1-mM CuCl_2_ influent case.

The breakthrough curve (BTC) of Cu^2+^ had an S shape with a sharp initial concentration increase for the three inflow concentrations, as shown in Figure 6. The results of cation displacement are shown in Figure 6d. The breakthrough onset occurred at 2.2 pore volume (PV).

The effluent Ca^2+^ concentrations first reached their peak values, then decreased to zero. This indicates the replacement of Ca^2+^ by Cu^2+^ through an ion exchange reaction in the AC matrix. Considering the 10-mM CuCl_2_ influent case as an example, the peak Ca^2+^ concentration occurred at 2.1 PV, which was close to the onset of the Cu^2+^ breakthrough (2.1 PV) and before the inflection point (i.e., the maximal concentration increases point) of the Cu^2+^ BTC (2.5 PV). A similar occurrence sequence of the peak Ca^2+^ concentration, followed by the onset of the Cu^2+^ breakthrough, was observed for the 1-mM and 5-mM CuCl_2_ influence cases. The results indicate that after displacing a major portion of Ca^2+^, which was originally in the AC samples, insufficient adsorption sites were available to contain Cu^2+^. Note that this portion was approximately 50%, corresponding to the peak Ca^2+^ concentration if a Gaussian distribution was assumed for the effluent Ca^2+^ concentration curve. The total amounts of displaced Ca(II) ion calculated using the curve are presented in Table 5. The ratios of the total Ca(II) ion concentration to the total adsorbed Cu(II) ion concentration were 47%, 74%, and 25%, which indicates that the Ca^2+^-Cu^2+^ ion exchange participated in a part of the adsorption process.

The curves of the electrical conductivity time evolution in Figure 6c reflected the cation exchange and adsorption processes. Considering the 10-mM Cu^2+^ inflow case as an example, the first electrical conductivity plateau of 2.1 PV coincided with the peak of effluent Ca^2+^ concentration, and its decline almost neutralized the effluent Cu^2+^ concentration, which results in a period of constant electrical conductivity (2.1–2.8 PV), as shown in Figure 6c. The second plateau value was obtained when the effluent Cu^2+^ concentration reached its maximum value of 4.2 PV.

The pH of the effluent solutions evolved from 6.7 to 7.0 to be close to that of the influent, as shown in Table 6. This process corresponds to the previously discussed chemical reactions. Minimal variations in the 1-mmol/L concentration and fluctuations around 6.7 were observed. This can be explained by the comparable pH values of the inflow fluid and the original DI water-saturated AC background liquid of 6.09 and 6.7, respectively. Similar degrees of decline were observed from approximately 6.7 to 5 within the ranges of 2–3.6 and 3.6–5 PV for the 5-mmol/L and 10-mmol/L cases, respectively. 

### 4.4. Adsorption Mechanism

Through the SEM–EDS methods, it can be observed that Cu^2+^ spots were evenly distributed on the surface of AC after adsorption. The pore size distribution measured using MIP methods remains unchanged before and after adsorption, indicating that no precipitation is formed, and the XRD results show no copper-related crystal is formed. The decrease in Ca^2+^ amount and increase in Cu^2+^ amount in EDS element analysis after contamination by copper ions indicate the ion exchange of metal ions. The increments of Ca^2+^ and Cu^2+^ ions in the effluent fluids in the column experiments further confirmed the process of ion exchange. In addition, the FTIR spectroscopy suggests that O–H, C=C, and C=O participated in the adsorption process. These function groups indicated that surface complexation could be one of the adsorption mechanisms of AC.

### 4.5. Typical SIP Responses

**Figure 7:**
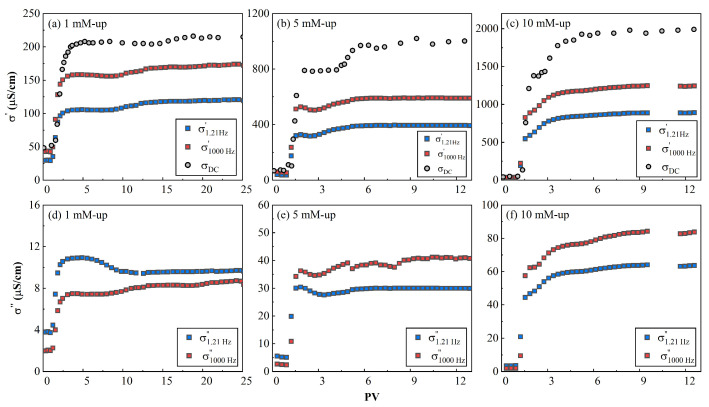
Evolution of the (**a**–**c**) real and (**d**–**f**) imaginary conductivities with time of the column at upper part in three different concentration solutions at 1.21 Hz and 1000 Hz. Note: the *x*-axis scale used for the 1-mM CuCl_2_ case is different from that used for the other cases.

**Figure 8:**
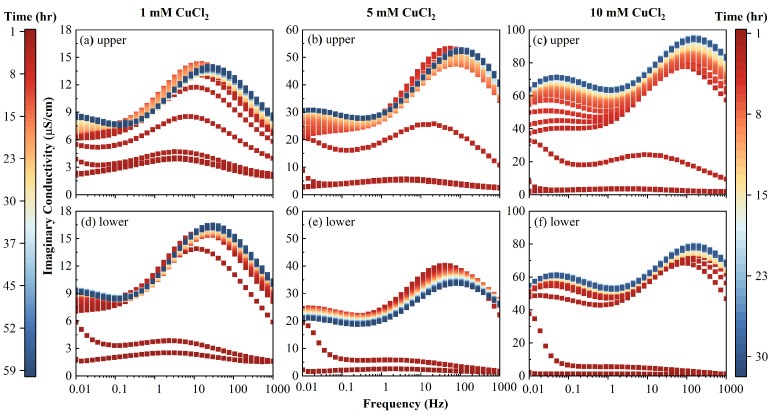
Evolutions of imaginary conductivities versus time measured of the column at (**a**–**c**) upper part using electrodes 1–2, and (**d**–**f**) lower part using electrodes 3–4. Time scale of 60 h for 1 mM and 30 h for 5 and 10 mM.

**Figure 9:**
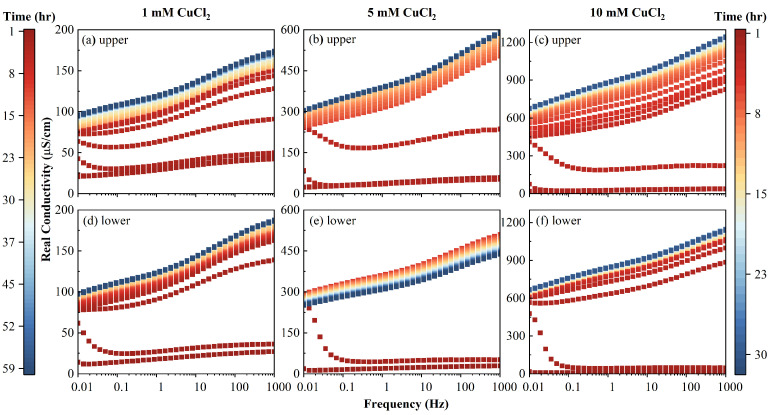
Evolutions of real conductivities versus time measured of the column at (**a**–**c**) upper part using electrodes 1–2, and (**d**–**f**) lower part using electrodes 3–4. Time scale of 60 h for 1 mM and 30 h for 5 and 10 mM.

The evolution of $\sigma^{\prime }$ and $\sigma^{\prime \prime }$, respectively, is plotted (Figure 7) at 1.21 Hz and 1000 Hz, where the peak responses of relaxation were not close. The typical SIP responses for three different contamination solutions are shown in Figure 8 and Figure 9.

It can be seen that the value of real conductivity increased with the injection of CuCl_2_ fluids (Figure 7); that is, with the increase of salinity. After the first PV, the real conductivity ($\sigma^{\prime }$) rapidly increased to a steady-state value, roughly from the second to the third PV (Figure 7). Afterwards, the real conductivity steadily reached a plateau value. The increase of the inflow salinity (primarily from the CuCl_2_ solutions) added more ions, which endowed the entire porous media with more conductivity. This trend was similar to that of the DC conductivity, as the real part is dominated by the pore fluid conductivity [28]. The real component can be written as:

(11) $$\sigma^{\prime }(\omega )=\frac{1}{F}\sigma_{w}+\sigma_{s}(\omega )$$

where $\sigma_{s}(\omega )$ denotes the surface conductivity [61] and F is the intrinsic formation factor.

Note that $\sigma_{s}(\omega )$ corresponds to the EDL and comprised two parts. The first part is from the frequency-independent diffuse layer, and the second one is from the frequency-dependent Stern layer [62]. The real part ($\sigma^{\prime }$) had a temporal pattern, which indicates that the ion exchange process occurred before the chemical reaction reached equilibrium. It can be deduced that $\sigma^{\prime }$ can be used as an alternative method to replace the outflow conductivity and provide more real-time feedback on the inflow process.

The clean initial AC imaginary conductivity had a single peak around 3 Hz. With the injection of inflow fluid, $\sigma^{\prime \prime }$ transformed from unimodal distribution into a bimodal one, and when the PV increased, the peak frequencies shifted to high frequencies, as shown in Figure 8. At a steady state, the characteristic frequencies at the two peaks increased with the increase of the inflow salinity, as shown in Figure 8. For example, the peak frequencies increased from 30 to 106, then to 147 Hz, and from a value less than 0.01 to 0.01, then to 0.05 Hz, for those two peaks, respectively. When the PV increased, the magnitude of the imaginary conductivity increased for the two peaks. It can be seen from Figure 7 that the magnitude of $\sigma_{1000\mathrm{Hz}}^{\prime \prime }$ was lower than that of $\sigma_{1.21\mathrm{Hz}}^{\prime \prime }$ at a concentration of 1 mM, while that of $\sigma_{1000\mathrm{Hz}}^{\prime \prime }$ exceeded that of $\sigma_{1.21\mathrm{Hz}}^{\prime \prime }$, with an inflow salinity greater than 5 mM. This phenomenon demonstrates that Cu^2+^ was mainly adsorbed and distributed in macrospores (1.21 Hz) at low salinity, as the value of imaginary conductivity reflected the polarizable charges on the surface of the porous medium.

It can be observed that $\sigma^{\prime \prime }$ increased with the PV for most of the inflow concentrations and upper/lower portions. The only exception was the case of the 5 mM of the lower column portion shown in Figure 8, where $\sigma^{\prime \prime }$ first increased until 2 h, then decreased. With a continuous influent injection, the adsorbed Cu^2+^ content continuously increased. This led to the increase of the number of polarizable units, which is due to the fact that the Cu^2+^ adsorbed the AC macropores. A detailed discussion on the latter phenomenon is presented in Section 4.6. A further injection of Cu^2+^ can lead to a plateau in the magnitude of $\sigma^{\prime \prime }$ and even to the decrease of $\left|\sigma^{\prime \prime }\right|$. This was possibly due to the connection of polarizable units or the diffuse layer between the AC macropores, which neutralized the dipoles comprising the EDL.

### 4.6. Cole–Cole Models

It can be seen from Figure 8 and Figure 9 that the SIP signals of the column at upper part (1–2) and lower part (3–4) show similarity, and the upper part (1–2) exhibits better performance, which is gradual. This is due to the fact that the upper part of the column is slowly infiltrated by the inflow solutions compared to the lower part. Therefore, the signals in the upper part (1–2) were employed to fit the double Cole–Cole model. The fitting results of lower part (3–4) are plotted and discussed in the Supplementary Materials.

**Figure 10:**
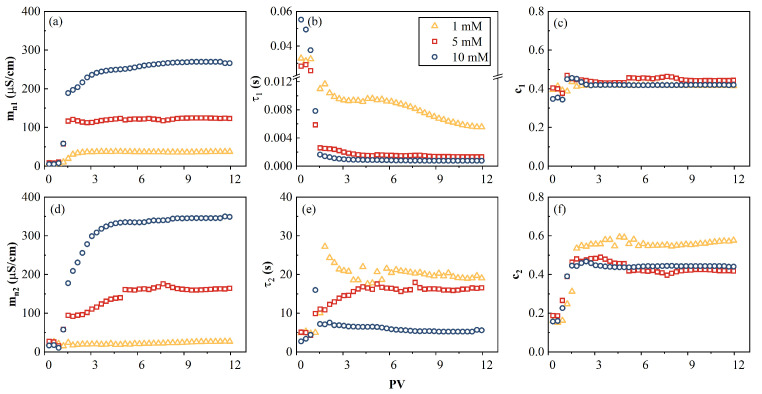
Parameters of the double Cole–Cole model function of time. (**a**,**d**) the normalized chargeability ($m_{n}$), (**b**,**e**) the relaxation time (τ), and (**c**,**f**) the exponent c at high and low peak frequencies, respectively.

The time evolution of the fitting parameters for the double Cole–Cole model (Equation (2)) is shown in Figure 10. The normalized chargeability, which represents the polarization magnitude, first increased with the inflow of CuCl_2_ solution, and then became almost stable after 3–7 PVs, as shown in Figure 10a,d. This indicates the saturation of Cu^2+^ adsorption on AC. It is also deduced that the Cu^2+^ BTCs reached the inflow concentration (C_0_) after 4–8 PVs, which were more delayed than the $m_{n}$, $\sigma^{\prime \prime }$, and $\sigma_{dc}$ curves. In addition, $m_{n}$ increased with the increase of the pore fluid conductivity, which is consistent with the results obtained by previous studies [18,34]. The relaxation times $\tau_{1}$ and $\tau_{2}$ first decreased and reached equilibrium within 9–12 PVs. Similar c trends suggested a similar broadness of the relaxation time distribution in the three inflow solutions.

### 4.7. Discussion

#### 4.7.1. The Normalized Chargeability vs. Content

**Figure 11:**
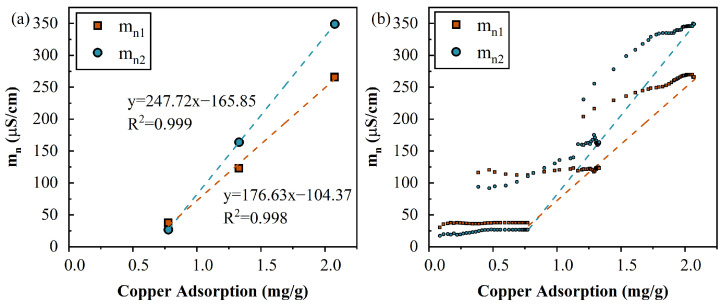
(**a**) Relationships between the copper adsorption capacity and normalized chargeability, $m_{n1}$ and $m_{n2}$ (the dotted line shows the linear fitting results). (**b**) The dynamic evolution process of $m_{n1}$, and $m_{n2}$ versus the copper uptake at each concentrations.

The relationship between $m_{n}$ and the adsorbed capacity at three salinities is shown in Figure 11. Figure 11a reflects the relationship between the adsorption capacity and the normalized chargeability when the adsorption reaches equilibrium. It can be seen that (1) the normalized chargeability ($m_{n1}$) at equilibrium (Figure 11a), which represents the content of polarizable units at the pore scale (1.7–0.6 µm) at equilibrium, was linearly proportional to the adsorbed Cu(II) (= 0.998). Similarly, the normalized chargeability ($m_{n2}$), which represents the content of polarizable units at the macrospore scale (99–54 µm), was also linearly proportional to the adsorbed Cu(II) ($R^{2}$= 0.999). The linearity validated that the chargeability revealed the adsorbed Cu^2+^ content at macrospores. In addition, this linearity was reported for the processes of cations adsorbed on soil, clay, and sand [34,63]. (2) The Cu^2+^ uptakes at two scale pores were simultaneous (Figure 11a), which indicates that when the influent concentration is increased, the distribution of the adsorbed Cu^2+^ between two scales pores becomes constant. (3) The time evolution of $m_{n1}$ and $m_{n2}$ at t=4 PV (shown in Figure 10a,d) further corroborated the aforementioned observation, as the relative positions of $m_{n1}$ and $m_{n2}$ were maintained (i.e., $m_{n1}$ > $m_{n2}$) and the chargeability at a high adsorbed Cu^2+^ content was high.

Figure 11b presents the evolution of the amount of adsorbate with the normalized chargeability under the three concentrations, which exhibits the effect of ion strength on $m_{n}$ during the adsorption process compared to Figure 11a. It can be seen that at the same adsorbed Cu^2+^ content, the normalized chargeability at a high influent conductivity was also high (Figure 11b). These mechanisms were attributed to the influence of the ionic strength of the pore fluid on the chargeability and the delayed Cu^2+^ adsorption equilibrium or the early attainment of the $m_{n}$ plateau value. 

#### 4.7.2. Calculated Pore Sizes

**Figure 12:**
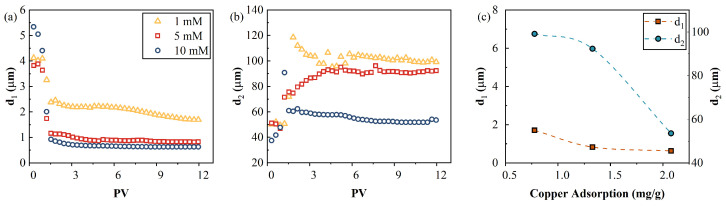
(**a**,**b**) Two scale pores calculated using the Cole–Cole model as a function of PV, and (**c**) relationship between the pore sizes ($d_{1}$ and $d_{2}$) and the copper adsorption content at equilibrium. Note that the dotted lines are an eye guide.

The characteristic polarizing unit size can be calculated using the Schwartz equation (Equation (3)) with a CuCl_2_ diffusion coefficient of 1.290 × 10^−9^ m^2^/s [64] at 25 °C. The average pore sizes of the imaginary conductivities at low frequencies were approximately in the ranges of 100–110, 80–90, and 53–60 µm for the 1, 5, and 10 mM influents, respectively. This falls into the average pore size range of 100 μm measured by MIP, as shown in Figure 3. When the calculated Cu^2+^ increased, the size of the characteristic polarizing units ($d_{2}$) decreased from 99 µm to 54 µm, as shown in Figure 12c. No crystals were observed by SEM. Therefore, the characteristic polarizing unit should be the pores with adsorbed Cu^2+^. Thus, the decreased $d_{2}$ indicates that the adsorbed Cu^2+^ migrated to small pores when the Cu^2+^ influx increased. Similarly, the calculated average pore sizes (.$d_{1}$.) of the contaminated AC were 2, 0.8, and 0.6 µm at high frequencies of imaginary conductivities for the 1, 5, and 10mM influents, which is consistent with the average pore size of 1 μm measured by MIP in Figure 3. The decrease of $d_{1}$ can also be attributed to the migration of adsorbed Cu^2+^ into small pores. In addition, the shrinkage of the electric diffuse layer caused by the increased ionic strength of the pore fluid may lead to the disconnection of the electric diffuse layer of adjacent pores and, consequently, a reduced polarizable unit size. The decrease of the size (corresponding to the characteristic increase of the frequency) with an increased fluid salinity is consistent with previous studies [65,66,67].

## 5. Future Outlook

Real groundwater contaminated by mining operations is often a multi-ion system [68], as opposed to the single-cation system analyzed in this study. The behavior of the ions may differ from that of the single-ion system. (1) In a multi-component system, the competitive adsorption effects will result in reducing the Cu^2+^ uptake. (2) In general, the adsorbents obey an adsorption selectivity sequence or lyotropic series [69]. For instance, Xue et al. [70] conducted batch experiments to study the removal characteristics of multi-ion by basic oxygen furnace slag, and the selectivity for adsorption isotherms follows the order Cu > Cd > Pb > Zn. Park et al. [71] evaluated the adsorption of multi-element onto sesame straw biochar with the selectivity sequence of Pb > Cu > Cr > Zn > Cd. (3) The pH will play an essential role in multi-ion systems as the proton can also compete with the adsorption [72]. Consequently, when studying the influence of multi-ion adsorption on the SIP signal, the above factors should be considered.

## 6. Conclusions

This study demonstrates the ability of SIP measurements to monitor the adsorption process on AC. The batch experiments and chemical analysis were conducted to study the adsorption performance. Three concentration cases of column experiments were performed. The following conclusions were drawn: 

(1) The adsorption isotherms of copper on AC follows the Freundlich model. The adsorption kinetics both follow PFO and PSO models. The adsorption thermodynamics demonstrates that the process is spontaneous and endothermic.

(2) The real and DC conductivities of effluents with 3 Cu^2+^ inflow concentrations showed similar trends. The electrical conductivity curves that had a temporary plateau reflected the cation exchange and adsorption processes. 

(3) The normalized chargeability was proportional to the copper adsorption capacity, at either the micropores or mesopores. During the adsorption process, the distribution of adsorbed Cu^2+^ between the mesopores and micropores was constant. 

(4) The calculated pore sizes at the 1-µm and 100-µm scales from the relaxation time of the SIP measurements were consistent with the average pore size obtained from the SEM and MIP tests. The observed decrease of the calculated size was attributed to the migration of adsorbed Cu^2+^ into small pores. 

(5) SIP method is a viable emerging monitoring technique for monitoring the copper adsorption onto AC process, and is able to shed light on the content, signature size, and the affiliation form of Cu^2+^.

## Figures and Tables

**Table 1:** Fitting parameters of Langmuir and Freundlich isotherm models.

Isotherm Models	Parameters	Temperature		
		298 K	308 K	318 K
Langmuir	q_m_ (mg g^−1^)	1.28	1.354	2.023
	K_L_ (L g^−1^)	0.02332	0.11145	0.08725
	R^2^	0.941	0.600	0.793
Freundlich	n	3.556	5.569	5.484
	K_F_ (L g^−1^)	0.214	0.482	0.689
	R^2^	0.968	0.939	0.997

**Table 4:** Thermodynamics parameters of Cu(II) adsorption on AC.

	Δ*G* (kJ mol^−1^)	Δ*H* (kJ mol^−1^)	Δ*S* (J mol^−1^K^−1^)
298 K	−6.46	46.24	177.41
308 K	−8.76		
318 K	−9.99		

**Table 5:** Calculated amount of Ca^2+^ displaced by Cu^2+^.

	Concentration	Ca (mg/g)
Parameters		
1 mmol/L		0.226
5 mmol/L		0.616
10 mmol/L		0.316

**Table 6:** Parameters of different concentrations of inflow CuCl_2_ solutions.

	Concentration	1 mmol/L	5 mmol/L	10 mmol/L
Parameters				
DC conductivity (μS/cm)		221	983	2040
pH		6.092	5.308	5.143
Concentration (mg/L)		62.56	312.7	625.8

**Figure A1:**
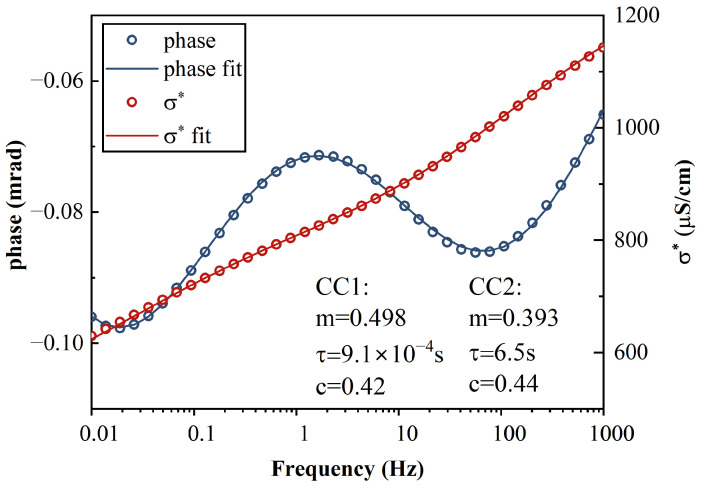
Example of the fitting results and SIP responses at 10 mM equilibrium. Two sets of Cole–Cole parameters are presented.

## Data Availability

Data sharing not applicable.

## Supplementary Materials

The following supporting information can be downloaded at: https://www.mdpi.com/article/10.3390/toxics11030221/s1.

## Appendix A

An example of the 10-mM concentration fitting results is shown in Figure A1, where the basic six Cole–Cole parameters are presented. The algorithm was coded using the Python language and modified using the pyGIMLi package, which is a Python-based open-source library for modeling and inversion. The open-source code for fitting SIP bimodal signals is available on the GitHub repository: https://github.com/bluspi/The-double-cole-cole-model-of-SIP-based-on-pygimli (accessed on 1 January 2023). It is recommended to follow the steps and to clone the repository to your system. In addition, a simple example for the simulation of multiple sets of SIP data is provided.
