# Supplementary material for: Investigation of Cu Adsorption and Migration with Spectral Induced Polarization in Activated Carbon
Source: Toxics. 2023 Feb 26;11(3):221. doi: 10.3390/toxics11030221 (PMC10057908; doi:10.3390/toxics11030221)
Supplement: Supplementary file 1 [file toxics-11-00221-s001.zip › toxics-2212989-supplementary.pdf]

Supplementary Materials

Overview

In the supplementary material, fitting results of the SIP signals of lower column portion (3-4) are provided.

S1. Cole–Cole models

S1.1 The Cole-Cole parameters vs. time

The time evolution of the fitting parameters of lower part (3-4) for the double Cole-Cole model is shown in Fig. S1. It can be seen that the parameters of lower portion exhibit similar trend to the upper portion's. However, since the lower portion is immediately infiltrated by the inflow solution at the beginning of the experiment, the normalized chargeability m_{n1}, m_{n2} shown in Fig. S1a and d reach equilibrium after 1.5 PVs, which is faster than the conditions in upper portion. This is corresponding to the faster saturation of Cu^{2+} adsorption on lower portion. The relaxation times τ_1 and τ_2 decreased and reached equilibrium within 9–12 PVs. Similar c trends suggested a similar broadness of the relaxation time distribution in the three inflow solutions.

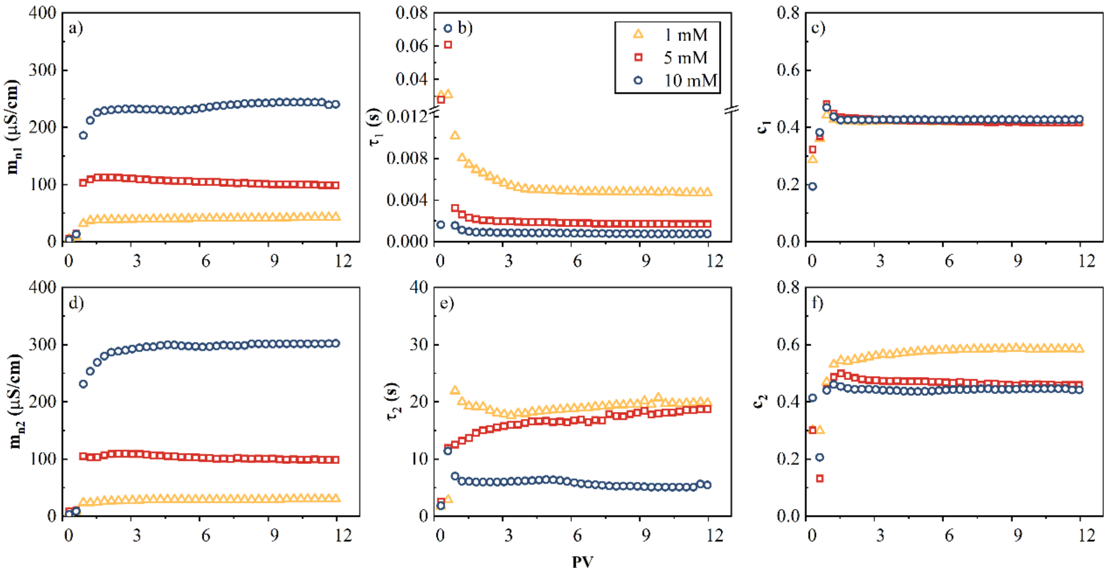

Figure S1. Parameters of the double Cole–Cole model function of time. (a) and (d) the normalized chargeability (m_n), (b) and (e) the relaxation time (τ), (c) and (f) the exponent c at high and low peak frequencies, respectively.

S1.2 The normalized chargeability vs. content

The relationship between m_n and the adsorbed capacity at three salinities is shown in Fig. S2. Fig. S2a reflects the relationship between the adsorption capacity and the normalized chargeability when the adsorption reaches equilibrium. It can be seen that (1) m_{n1} at equilibrium, which represents the content of polarizable units at the pore scale (1.6–0.6 um) at equilibrium, was linearly proportional to the adsorbed Cu(Ⅱ) (R^2 = 0.951). Similarly, the normalized chargeability (m_{n2}), which represents the content of polarizable units at the macrospore scale (101–53 um), was also linearly proportional to the adsorbed Cu(Ⅱ) (R^2 = 0.926). The linearity validated that the chargeability revealed the adsorbed Cu^{2+} content at macrospores. (2) The Cu^{2+} uptakes at two scale pores were simultaneous (Fig. S2a), which indicates that when the influent concentration increased, the distribution of the adsorbed Cu^{2+} between two scales pores becomes constant.

Figure S2b presents the evolution of the amount of adsorbate with the normalized chargeability under the three concentrations. The evolution of processes show nearly parallel to copper adsorption in all conditions, indicating that SIP responses reach equilibrium shortly after the influx inflow. This phenomenon reflect the influence of ion strength of the pore fluid on the normalized chargeability.

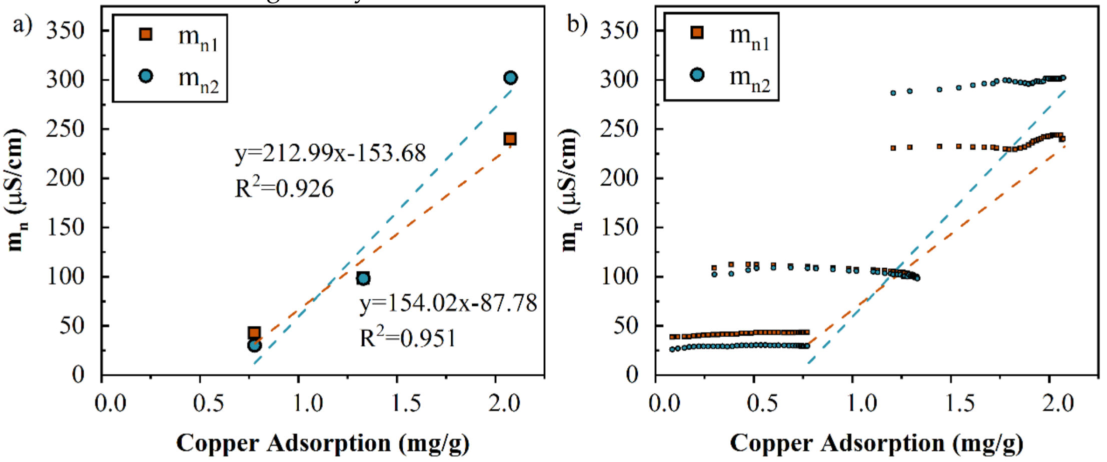

Figure S2. a) Relationships between the copper adsorption capacity and normalized chargeability, m_{n1}, and m_{n2} (the dotted line shows the linear fitting results). b) The dynamic evolution process of m_{n1} and m_{n2} versus the copper uptake at each concentrations.

S1.3 Calculated pore sizes

The calculated characteristic polarizing unit sizes using the Schwartz equation (Eq. 3) with a $CuCl_2$ diffusion coefficient of 1.290 × 10^{-9} m^2/s are shown in Fig. S3. Figure S3a and b reflect the evolution of pore sizes with PV. The average pore sizes of d_2 were approximately in the ranges of 95–110, 80–100, and 50–60 μm for the 1, 5, and 10 mM influents, respectively. This falls in the average pore-size range of 100 μm measured by MIP, as shown in Fig. 3. Similarly, the calculated average pore sizes (. d_1 .) of the contaminated AC were 1.7, 1, and 0.8 μm for the 1, 5, and 10mM influents, which is consistent with the average pore-size of 1 μm measured by MIP.

Figure S3c show the relationship between the copper capacity and the pore sizes (d_1 and d_2). When the copper capacity increased, the size of the characteristic polarizing units (d_2) decreased from 101 μm to 53 μm, as shown in Fig. 12c. No crystals were observed by SEM. Therefore, the characteristic polarizing unit should be the pores with adsorbed Cu^{2+}. Thus, the decreased d_2 indicates that the adsorbed Cu^{2+} migrated to small pores when the Cu^{2+} influx increased. The decrease of d_1 can also be attributed to the migration of adsorbed Cu^{2+} into small pores. In addition, the shrinkage of the electric diffuse layer caused by the increased ionic strength of the pore fluid may lead to the disconnection of the electric diffuse layer of adjacent pores, and consequently, a reduced polarizable unit size.

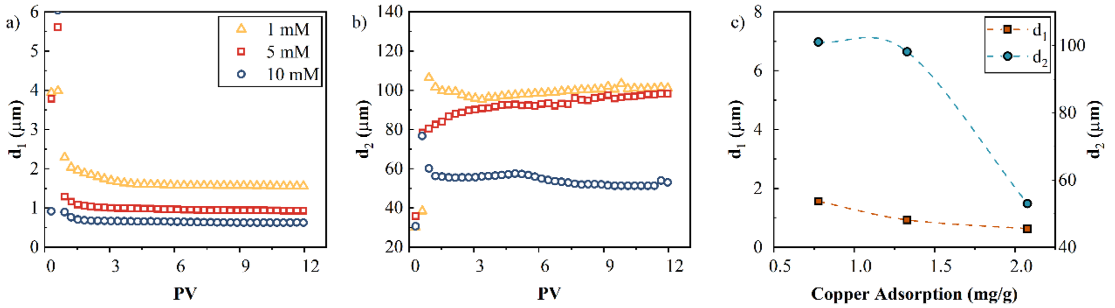

Figure S3. (a-b) Two scale pores calculated using the Cole–Cole model as a function of PV, and (c) relationship between the pore sizes (d_1 and d_2) and the copper adsorption content at equilibrium. Note that the dotted lines are an eye guide.
